# Evaluating the implementation of community engagement guidelines (EVALUA GPS project): a study protocol

**DOI:** 10.1136/bmjopen-2022-062383

**Published:** 2023-02-23

**Authors:** Viola Cassetti, María Victoria López-Ruiz, Marta Domínguez, Alba Gallego-Royo, Ana María García, Vicente Gea-Caballero, Catalina Nuñez, Joan Josep Paredes-Carbonell, Luis Angel Pérula-De Torres, Marina Pola-Garcia, EvaluAGPS Research Group, Aldecoa Landesa Susana, Carmen Belen Benedé Azagra

**Affiliations:** 1 Independent Researcher, Castellón, Spain; 2 Government of Andalusia Andalusian Health Service, Sevilla, Spain; 3 Maimonides Biomedical Research Institute of Cordoba (IMIBIC), Reina Sofia University Hospital, University of Cordoba, Cordoba, Spain; 4 Health Research Institute Aragón, Zaragoza, Spain; 5 Servicio Aragones de Salud, Zaragoza, Spain; 6 University of Valencia, Valencia, Spain; 7 CIBERER, Valencia, Spain; 8 Faculty of Health Science, Valencian International University, Valencia, Spain; 9 Research Group in Community Health and Care SALCOM, Valencian International University, Valencia, Spain; 10 Health Promotion Service, General Directorate of Public Health, Balearic Islands, Spain; 11 La Ribera Health Department, Primary Care Management, Valencia, Spain; 12 Teaching Unit of Family and Community Medicine, Health District of Cordoba and Guadalquivir, Cordoba, Spain

**Keywords:** public health, protocols & guidelines, qualitative research, social medicine

## Abstract

**Abstract:**

**Introduction:**

The EVALUA GPS project aims to evaluate the impact of the implementation of the National Institute for Health Care and Excellence (NICE) guideline ‘Community engagement: improving health and well-being and reducing health inequalities’ adapted to the Spanish context.

**Methods and analysis:**

Phase I: A tool will be designed to evaluate the impact of implementing the recommendations of the adapted NICE guideline. The tool will be developed through a review of the literature on implementation of public health guidelines between 2000 and 2021 and an expert’s panel consensus. Phase II: The developed tool will be implemented in 16 community-based programmes, acting as intervention sites, and 4 controls through a quasi-experimental pre–post study. Phase III: A final online web tool, based on all previously collected information, will be developed to support the implementation of the adapted NICE guidelines recommendations in other contexts and programmes.

**Data collection and analysis:**

Data will be collected through surveys and semistructured interviews. Quantitative and qualitative data will be analysed to identify implementation scenarios, changes in community engagement approaches, and barriers and facilitators to the implementation of the recommendations. All this information will be further synthesised to develop the online tool.

**Ethics and dissemination:**

The proposed research has been approved by the Clinical Research Ethics Committee of Aragon. Results will be presented at national and international conferences and published in peer-reviewed open access journals. The interactive online tool (phase III) will include examples of its application from the fieldwork.

**STRENGTHS AND LIMITATIONS OF THIS STUDY**

- The mixed-methods (quantitative and qualitative) approach adopted in this research could support both researchers and participants to increase their knowledge and practice about community engagement in health programmes.
- Research on public health guidelines implementation and evaluation is limited, and evidence is needed to help bridge the gap between research, policy and practice.
- Engaging a variety of stakeholders from different backgrounds throughout the phases of the project strengthens the research project and the potential transferability of the study results.
- Researching community engagement during the COVID-19 pandemic is challenging but this project can support community programmes in these difficult times

## Introduction

 Engaging people and communities is central to the improvement of their health and well-being and to the reduction of health inequalities.^1^ According to the WHO,^2^ community engagement in health is essential to protect and improve populations’ health. Through community engagement, local people increase their decision-making capacities and trust among themselves, allowing them to influence the social determinants of health that affect them, to improve their health and that of their community.^3 4^ Nonetheless, despite increasing evidence on its importance,^5 6^ there is still a need to improve knowledge and practice about community engagement in health.^7^

In 2016, the National Institute for Health Care and Excellence (NICE), a UK institute dedicated to the development of evidence-based guidelines, related to both clinical and public health topics, reviewed the evidence on the effect of community engagement on the health and well-being of communities and on reduction of health inequalities, and provided recommendations for incorporating community engagement into health policies and interventions in the published NICE Guidance NG44.^1^ During 2017 and 2018 a collaborative project was carried out by a group of health-related professionals in Spain to adapt the NICE guideline NG44 to the Spanish context, the AdaptA GPS project.^8^ The project resulted in the first public health guideline included in GuíaSalud, the clinical guidelines catalogue of the Spanish Ministry of Health.^8^ At present, there is no evaluation of the implementation of the guidelines in the GuíaSalud catalogue: once developed, there is no evaluation of their impact on practice or health.

The project EvaluA GPS (from its Spanish acronym: Evaluating the Application of Health Promotion Guidelines) aims to evaluate the impact of the implementation of the Spanish adapted NICE guideline NG44, through the following specific objectives:

Objective 1: To develop an implementation and evaluation tool based on the recommendations of NICE guideline NG44, in order to identify changes which can improve community engagement in community-based health programmes carried out in different contexts.

Objective 2: To evaluate the impact of implementing the recommendations of NICE guideline NG44 on community engagement in a selection of community-based programmes.

Objective 3: To identify different implementation approaches according to different contexts.

This paper presents the research protocol for the project EvaluA GPS.

## Methods and analysis

**Figure 1:**
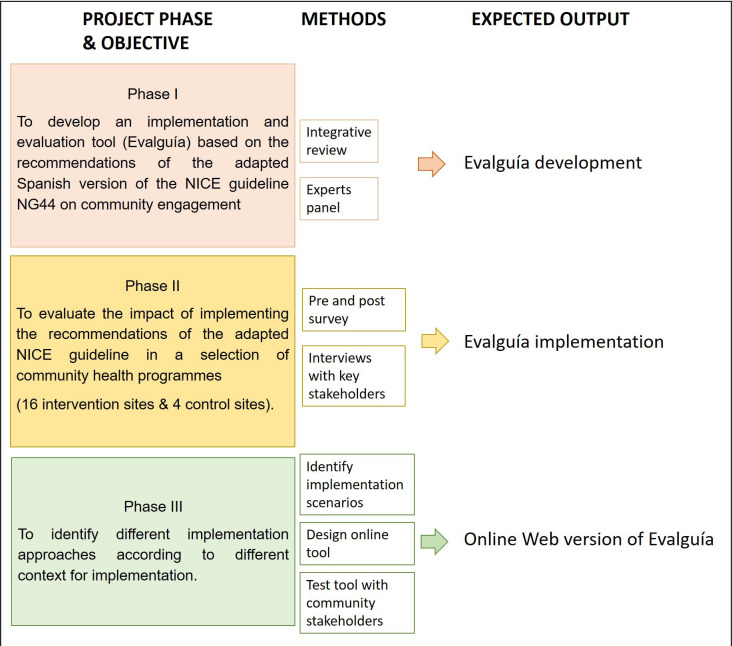
The three phases of EvaluA GPS project. Own elaboration. NICE, National Institute for Health and Care Excellence.

EVALUA GPS Project will be developed through three main phases, as illustrated in figure 1. Phase I will centre on developing a tool to evaluate the impact of the recommendations (objective 1). In phase II, the developed tool will be implemented in a selection of community-based programmes to explore changes in community engagement (objective 2). Phase III will then synthesise the information from the implementation phase, to develop an online tool to support the implementation of the adapted guidelines recommendations in different scenarios (objective 3).

### Phase I: development of the implementation and evaluation tool (Evalguía)

The implementation and evaluation tool will be developed using evidence from an integrative review of the literature on public health guidelines implementation^9^ and an expert panel using an adapted Delphi method.^10^

To develop the first version of the evaluation tool (Evalguia 0.1), an integrative review was conducted to identify the available literature on the implementation of public health guidelines.^9^ Integrative reviews use a systematic approach to search relevant articles about the topic of interest, and provide a critical analysis of the findings, often including a thematic synthesis approach, as it has been done in this case.^11^ A systematic search strategy combining key terms and synonyms related to ‘implementation’, ‘guidelines’ and ‘analysis’ or ‘evaluation’ was conducted using the databases PubMed, CINAHL, Web of Science and Scopus.

Only studies analysing and/or evaluating the implementation of public health guidelines were included. Three researchers identified the papers to be included in the review through screening titles and abstracts and data were analysed using a thematic synthesis approach.^12^ The review followed the ENTREQ (Enhancing transparency in reporting the synthesis of qualitative research) statement to structure and report the review process.^13^ The findings from the integrative review informed the development of the first version of the Evalguia tool (Evalguia V.0.1).

The Evalguia 0.1 will be tested through a panel of experts. This will be organised through an adapted Delphi method,^10^ through two rounds of review of the tool. It is defined as ‘adapted’ Delphi as each expert will provide individual feedback about the tool, but the analysis and consensus on the modifications to be made to the tool will be carried out by the EvaluA GPS research team, with an aim to include all comments provided by the experts.

To identify experts in community health interventions, each researcher of the EvaluA GPS team (composed of a total of 35 researchers) will be asked to suggest at least one potential participant, who will be independent to the project. A maximum of 60 experts will be identified prior to the first round of invitation, considering both geographical variability (experts from different regions of Spain) and roles (academic, practitioners, community workers, local organisations’ members) as selection criteria. No exclusion criteria will be considered. Once the list has been compiled, an online invitation will be sent by email to these experts, who, after expressing their interest in participating, will be sent an informed consent form, which they will have to sign and return.

All participating experts will then receive the Evalguía V.0.1 in a word document online, so that they can provide their feedback and suggestions directly in the word document. They will be asked to review it with a focus on content, language and design. After receiving comments from the first round of review, the research team will compile and discuss the proposed changes, and will finally select the changes to be made. The tool will then be modified accordingly and Evalguia V. 0.2 will be developed. Participants who have not submitted the revision will be excluded from the following round. Evalguia V.0.2 will then be sent again to the participating experts, requesting their final revision after the proposed changes.

Finally, after receiving the second round of reviews, the research team will discuss the proposed changes and modify the tool accordingly, trying to include them all where possible. This final version, Evalguia V.0.3, will therefore be considered as the final version of the tool to be implemented in phase II.

### Phase II: implementation

This phase aims to pilot-test the developed implementation and evaluation tool in a selection of community-based programmes, using a quasi-experimental pre and post study (objective 2). To select the programmes, researchers from the EvaluA GPS team will suggest ongoing community-based programmes, with an aim to identify a total of sixteen local programmes in four different Spanish regions to act as ‘interventions sites’. Four additional programmes will be selected to act as ‘control sites’ in only one of the regions (Aragon). The programmes will not be randomly assigned to become intervention or control sites, but deliberately selected for each group. All programmes will be selected with the following inclusion and exclusion criteria:

1. Inclusion criteria:
  1. The community-based programme should aim to improve community health or follow at least one of the five lines of actions of the Ottawa Charter for Health Promotion (‘1-building healthy public policy, 2-creating supportive environments, 3-strengthening community action, 4-developing personal skills, and 5-reorienting healthcare services towards promotion of health’).^14^
  2. The community should participate in at least two phases of the programme (health needs assessment, design, implementation, evaluation).
  3. The level of community engagement can be defined according to one of the next five participation levels: informing, consulting, cocreating decisions and actions, multiple and shared leadership and/or community control, as described in the NICE Guidance NG44.^1^
  4. The programme should have been ongoing for at least 1 year.
2. Exclusion criteria:
  1. The programme aims to promote individual health only, lacking a community-based health approach.
  2. The level of community engagement is limited to informing or consulting the community.

#### Intervention

All the 20 selected community-based programmes (intervention sites, n=16 and control sites, n=4) will receive the implementation tool (Evalguia V.0.3), in the form of a written document, which will include written instructions on how the tool should be implemented. The selected 16 interventions sites will also receive an implementation workshop developed ad hoc by the research team based on previous experience in the field of community-based actions and on the results from phase I, while the remaining four control sites will only receive the implementation tool which they will have to self-administer following the written instructions. This will allow to evaluate whether the implementation tool alone (without supplementary support from the workshop) improves community engagement in the selected community-based programmes, and to identify potential facilitators for the implementation.

In each intervention site, the workshop will be delivered to key stakeholders such as managers, front-line workers and community members involved in the projects. The research team will recommend that the workshop will be carried out in two sessions, and where possible on two different days or with a lunch break in between. However, the research team will need to adapt to the availability of the participating stakeholders. It will be recommended to have a maximum of 15 people attending, to facilitate the process and the group activities. In the first workshop session, after a general presentation of the project and the participants, we will define key terms included in the guidelines, such as community engagement, health assets, intersectoral work, vulnerable groups and empowerment, to ensure all participants will have a shared understanding of these concepts. Then the Evalguía tool will be presented and implemented, followed by a group discussion on the results. This will allow participating stakeholders to reflect how community engagement is being currently carried out in their programmes, and to identify areas for improvement. In a second session, an action plan aimed at improving community engagement in the project following the adapted guidelines recommendations will be elaborated. It is foreseen that the workshop will be held in person, although as a consequence of the COVID-19 pandemic these may have to be held online.

### Data collection

**Figure 2:**
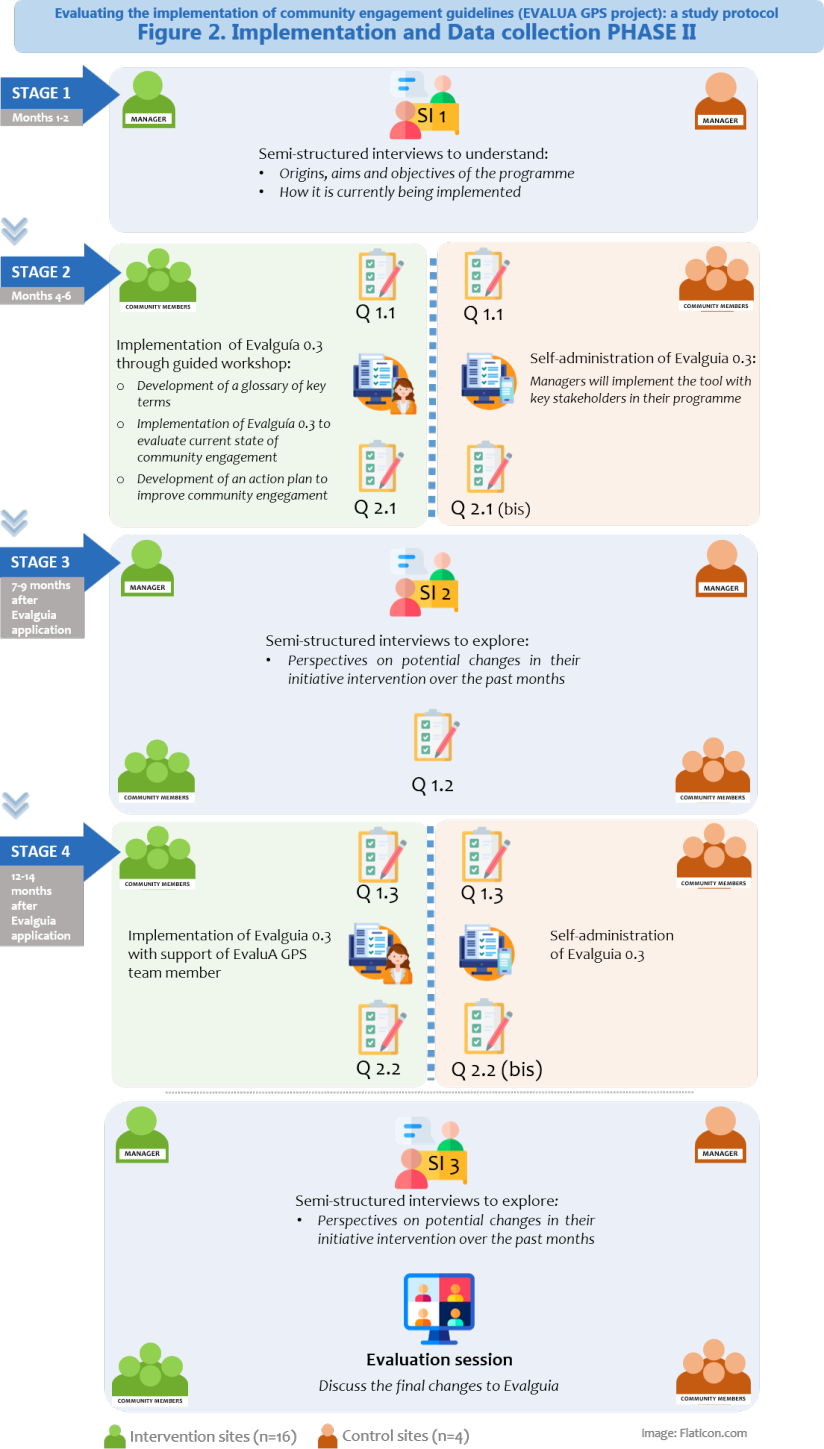
Data collection phase II. Own elaboration.

Data collection during the implementation process (phase II) will be structured into four stages, over a period of 15–18 months (figure 2).

In stage 1 (months 1–2), a semistructured interview (SI1) will be held with the programme’s managers (duration 60–90 min) to better understand the community-based programme, its origins, its aims and objectives and how it is being implemented and carried out daily.

In stage 2 (months 4–6), the Evalguia tool will be applied: 16 ‘intervention sites’ will be supported through the implementation workshop, while the 4 ‘control sites’ will only receive the tool. Prior to the application of the Evalguia tool, all the participating programmes (n=20) will answer an initial closed-answer questionnaire (Q1.1), which will explore their perspective on the community engagement approach currently being used in their programmes. The initial questionnaire will include questions about who is currently involved in the community-based programmes and who are the main decision-makers at each project stage (health assessment, design, implementation and evaluation). This information will provide a baseline as to what extent community members are engaged in the different stages of the selected community-based programmes, and it will allow comparison before and after the implementation of the Evalguia tool, to check its impact on community engagement in the programme. At the end of the implementation session, to evaluate the Evalguia tool and the workshop, participants from the intervention programmes (n=16) will answer a closed-answer questionnaire (Q2.1), exploring their opinion of the tool (15 min) and of the workshop itself. Participants in the control programmes will answer a questionnaire (Q2.1 bis) exploring their opinions on the tool only.

In stage 3 (7–9 months after the Evalguia application), participants of all 20 programmes will be asked to answer a short questionnaire on perceived changed in community engagement in their programmes (Q1.2), and a second semistructured interview (60 min) (SI2) will be carried out with programme managers to explore their perspectives on potential changes in their programme over the past months.

In stage 4 (12–14 months after the Evalguia application), all community-based programmes (n=20) will implement again the Evalguia tool in their own project (16 intervention sites will be supported by a EvaluA GPS team member, while the 4 control site will self-administer the tool), to check whether there have been changes in community engagement approaches. Stakeholders who participated in the Evalguia application will then answer a questionnaire on perceived changes in community engagement in their programmes (Q1.3), and a final questionnaire on the Evalguía tool (Q2.2 and Q2.2 bis for participants from the intervention and control programme respectively). A final semistructured interview (SI3) with the managers will be conducted (60 min) to discuss perceived changes in their programmes. To conclude, an online evaluation session will be organised with all the participants from the 20 programmes to discuss the final changes to Evalguia.

Written consent will be obtained from each stakeholder prior to data collection. Interviews will be audiorecorded and transcribed, and the intervention workshop as well as the application session in the control group will be audiorecorded. Then, transcripts, audio recordings and the action plans will be imported to NVivo V.12 software to support the qualitative analysis.

#### Analysis

The data collected through the fieldwork in phase II will be analysed to identify changes in community engagement approaches and other possible changes resulting from the implementation of the recommendations and/or of the workshops (such as organisational changes and changes in relationships). Data from the questionnaires will be analysed using descriptive statistics using SPSS version 27.0, to compare changes pre and post intervention in both the interventions and the control programme. Data from interviews and workshops will be analysed using a thematic analysis approach.^15^ The analysis will focus on synthesising similarities identified in the intervention and control programmes to identify different implementation scenarios where community engagement can be enhanced and allow the transferability of results to different contexts. Moreover, the analysis will identify (A) barriers and facilitators in the implementation of the recommendations and (B) strategies to overcome these barriers and promote facilitators, to support implementation in other contexts. Qualitative analysis will be conducted by two researchers separately, using NVivo software V.12 to aid the analytical process. Codes and themes will be then compared and synthesised together.

Both quantitative and qualitative data will contribute to answering the objectives of the study. Triangulation will strengthen the results of the study,^16^ as quantitative data will provide an assessment of changes in community engagement which could be attributed to the implementation of the guideline recommendations, allowing also to evaluate the impact of the workshops. These changes will be checked against codes and themes identified in the qualitative analysis. The qualitative analysis about contextual factors and barriers and facilitators to the implementation, together with quantitative data about the Evalguia tool will then set the basis to develop an online tool (phase III) to support the implementation of the adapted guidelines recommendations in other programmes and contexts, thus enhancing the transferability and applicability of the study results.

### Phase III: online tool development

The data collected through the implementation phase will then support the development of an interactive online tool. The tool will be structured according to different potential scenarios for implementation and will include examples from the field as evidence of good practice to improve community engagement. Importantly, the interactive web tool will be designed with lay and inclusive language. The online tool will be tested through a final online evaluation session with the stakeholders who participated in the application of Evalguia, to ensure its language and design are user-friendly and accessible for a lay audience.

### Patient and public involvement statement

EvaluA GPS Project is based on the findings of the previous research project, AdaptA GPS,^8^ in which community members from eleven local community-based programmes across Spain were involved to test the adapted NICE NG44 guidance. The EvaluA GPS protocol has been developed following their feedback on the need to simplify the language used in the recommendations and provide more practical examples on how to implement the community engagement recommendations. Community members are also key stakeholders in the EvaluA GPS project, as they will be involved in all stages of the project. In the initial phase, local organisations’ members will be invited to participate in the review of the Evalguia tool through the adapted Delphi method. Following this, in the implementation phase, community members will be recruited to participate in the workshops (intervention). In addition, community members will be invited to the final online evaluation session to consult them on how to improve the Evalguia tool in its online version.

## Ethics and dissemination

All participants will receive an information sheet detailing all the phases of the research project and will be informed of the objectives and characteristics of the study, as well as their voluntary participation and the possibility of leaving the study at any time during the research process. The intervention programmes will be informed that they will receive a workshop to implement the Evalguia, while the control programmes will be informed that they will receive the Evalguia and will have to implement it on their own. If they agree to participate in the study, participants will be asked to sign an informed consent form. Data confidentiality will be guaranteed in accordance with Spanish Organic Law 3/2018 on the protection of personal data and guarantee of digital rights, being analysed anonymously and described in aggregate form to avoid identification at an individual level. The research project has been approved by the Clinical Research Ethics Committee of Aragón, PI20/116.

The dissemination strategies include that study results will be presented at national and international conferences, as well as published in open access peer-reviewed journals. Moreover, the research team will develop a short animated video summarising the project,^17^ which will support presenting the research to lay people.

One of the goals of translational research is the translation of new approaches into a form amenable to widespread adoption and implementation.^18^ The EvaluA GPS research described here may enhance translational research, as it intends to facilitate the application of evidence-based recommendations for community-based health programmes, hence contributing to reducing the gap between research, policy and practice.^19^ At EvaluA GPS we aim to develop implementation scenarios to facilitate project design and evaluation. Moreover, it is hoped that generating more practice-based evidence in community engagement in health in Spain will strengthen and enhance good practices in community health programmes, contributing to promote the health of people and communities and reduce health inequalities.
