# Supplementary material for: Evaluating the implementation of community engagement guidelines (EVALUA GPS project): a study protocol
Source: BMJ Open. 2023 Feb 23;13(2):e062383. doi: 10.1136/bmjopen-2022-062383 (PMC9950881; doi:10.1136/bmjopen-2022-062383)
Supplement: Supplementary file 1 [file bmjopen-2022-062383.reviewer_comments.pdf]

PEER REVIEW HISTORY

BMJ Open publishes all reviews undertaken for accepted manuscripts. Reviewers are asked to complete a checklist review form (**http://bmjopen.bmj.com/site/about/resources/checklist.pdf**) and are provided with free text boxes to elaborate on their assessment. These free text comments are reproduced below.

ARTICLE DETAILS

**TITLE (PROVISIONAL)**	Evaluating the implementation of community engagement guidelines (EVALUA GPS project): a study protocol
**AUTHORS**	Cassetti, Viola; López-Ruiz, María Victoria; Domínguez, Marta; GALLEGO ROYO, ALBA; García, Ana; Gea-Caballero, Vicente; Nuñez, Catalina; Paredes-Carbonell, Joan Josep; PÉRULA DE TORRES, LUIS ÁNGEL; Pola-Garcia, Marina; EvaluAGPS Research Group, EvaluAGPS Research Group; Benedé. Azagra, Carmen Belen

VERSION 1 – REVIEW

**REVIEWER**	Leavy, Justine<br>Curtin University, School of Public Health
**REVIEW RETURNED**	31-Mar-2022

**GENERAL COMMENTS**	BMJ Open-2022-062383<br><br>Evaluating the implementation of community engagement guidelines (EVALUA GPS project): a study protocol.<br><br>General comments. Thank-you for asking me to review the manuscript titled as per above and describes the proposed protocol that aims to evaluate the impact of the implementation of the Spanish adapted NICE guidelines. Overall, it is an interesting and under published area. However, I have concerns that a protocol should describe prospective work that has not been commenced and in this paper the integrative review has already been completed and is under review for publication. It is my understanding that a protocol describes work to be undertaken not work that is partly complete. Perhaps the Phase 1, stage 1 needs to be removed and the manuscript re-written to reflect the work to be undertaken. I assume the Editor-In-Chief will make a decision on this. However, the comments below are provided to strengthen your paper. All the best.<br><br>Abstract<br><br>Methods page 3 of 13, line 20 suggest you include the proposed dates for the literature review e.g. 2012-2022 if this is to stay in the paper.<br><br>Data collection and methods: line 36 suggest you add exploring changes in community engagement intervention to set the context early in the paper.<br><br>Introduction:

Could you briefly explain the NICE guidelines and what they cover for readers who may not be familiar with these?

Page 5 of 13, line 13-14 Objective 1.2 again I would add community engagement health interventions here so the focus clear, otherwise it could mean any health intervention.

Methods and analysis: page 5 of 13 line 26 – is this an analysis of the literature or a review of the literature? I am unsure what an integrative review is exactly. Can the authors provide a definition of how an integrative review is different from a narrative or systematic literature review or a scoping review?

Phase 1: part one: integrative review

This is where I have an issue with the manuscript it describes a '….review will be conducted …..' page 5, line 50. Phase 1: part 2 Page 6 of 13, line 25-27 suggests the review has been completed and under consideration for publication.

Was a systematic search strategy used, e.g. are you following any protocol (eg PRISMA) to ensure rigour?

Page 5 line 56 you comment on peers but did you also engage a reference librarian to refine your search terms? Can the authors comment on this?

Page 6 first paragraph the – inclusion criteria is cumbersome, I suggest you simply state criteria 3 and use and/or to include criteria 1 and 2, in one sentence.

How will you check inter-rater reliability? Will you have a checklist or randomly check selections or exclusions? PRISMA protocol will help with this.

Page 6, line 15 check if ENTREQ needs to be fully described here.

Phase 1 part two: expert panel through adapted Delphi method

Page 6, line 33, paragraph2 – consider including n = X values to identify how many experts you will recruit. Could the authors comment on using a using snowball method versus selecting experts known to the researchers? See: Naderifar, M., Goli, H., & Ghaljaie, F. (2017). Snowball sampling: A purposeful method of sampling in qualitative research. Strides in Development of Medical Education, 14(3).

Page 6 , line 45 - how will experts review the tool (Evalguia 1.0) sent a link, then surveyed or interviewed? Can you add some detail here?

Can you authors provide more detail on the 'adapted methodology' you are using and how it differs from traditional Delphi processes? Will round one findings be collated with comments included in round two data collection tools, as is typical for Delphi model. How will consensus be reached? Who will decide, if the experts disagree?

Phase II: implementation

	Page 7 , line 9 - How will the community based interventions be recruited? Are they individual interventions that happen to be starting at the same time or will some be underway already? How will you ensure you capture various types of community based interventions and in various settings – this is especially important if you hope to reach the analysis goals listed on page 9 (Analysis section)<br><br>Page 7, line 48 - How will you randomise which of the 20 interventions are control interventions (n=4) and which get the full implementation of the tool (n=16)? Can the authors provide some more details<br><br>Intervention<br><br>Page 7 line 57, 58 over to page 8 the final sentence in paragraph 1 is incomplete/does not make sense.<br><br>Page 8, line 9 you say 'in' two different days it should read on two different days. You may like to have a native English language speaker proof read your paper.<br><br>Page 8, line 19, paragraph 2, when explaining the workshop, you state "the Evalguia tool will be implemented" do you mean introduced? Can you please clarify this.<br><br>Data collection<br><br>Is the implementation process (Phase II)? If so, please indicate this in paragraph 1<br><br>Page 8, line 33 this sentence is awkward consider re-writing 'Written consent will be obtained from each stakeholder prior to data collection commencing.'<br><br>Page 8, line 41 – consider n=16 intervention groups and n = 4 control groups for clarity and consistency throughout the paper.<br><br>How will data from community engagement surveys support the development of the tools (As indicated on page 9 – Phase III section) – what questions will you ask? Consider outlining your Domains of inquiry here<br><br>Page 8, line 56 Would the authors consider semi-structured interviews as better fit for the interviews discussed in stage 1. These interviews are exploring perspectives on potential changes so some structure e.g. around barriers and enablers for the Evalguia tool might be better than unstructured interviews. Can you provide a rationale for unstructured interviews?<br><br>Will qualitative data be recorded, transcribed and will there be any member checking?<br><br>You state In Stage 3 - 7-9 months after the Evalguia implementation - Do you mean after the workshops and/or intervention has commenced?<br><br>Data collection includes: Community engagement surveys at 1 month, 4 months and 12 months (n=16 interventions) for all who participated in the workshops (n=15) as well as interviews with

	intervention manager (n=16) at 1 month, 4 months and 12 months. In addition, an evaluation session/workshop (n=20) and final survey (n=20). Have I understood this correctly? Perhaps include this as a Figure<br><br>Page 9 line 5 – who exactly are these stakeholders?<br><br>Phase III: Online tool development<br><br>Page 9, line 16 -17 - how will data from community engagement surveys support the development of the online tool – what questions will you ask? Perhaps outline the variable sets in the Community Engagement Survey tool in Phase II of the study<br><br>Analysis The analysis focus highlights the need for semi-structured interviews rather than unstructured, as described.<br><br>What package will you use to analyse the quantitative data and similarly what will be used to analyse the interview qualitative data.<br><br>Page 9, line 47 onwards – is there any opportunity for triangulation of the data here? Can the authors please comment<br><br>Page 9, line 57 You state you aim to enhance translational research – can you give an example or two?<br><br>General comments for review<br><br>Check if headings need be italicised to differentiate from main titles throughout? Eg Should this be italicised to differentiate from main titles? The headings confuse the flow of this article. (i.e. Methods, Phase II, Intervention).<br><br>Page 7 a full stop is required at point d.<br><br>Page 8, line 55 - repetition of words in paragraph 4 “asked to answer again to answer” needs deleting<br><br>National Institute for Health and Care Excellence (NICE) might need to also be written out in full for readers.<br><br>Figure 1 was hard to read in the version I received. Please update for readability as it looks incomplete with the arrows on the right and all the black space. Is there information missing here?

**REVIEWER**	Turin, Tanvir<br>University of Calgary, Department of Family Medicine, Cumming School of Medicine,
**REVIEW RETURNED**	11-Apr-2022

**GENERAL COMMENTS**	This a protocol of study which aims to evaluate the impact of the implementation of the NICE guideline informed Community Engagement in a selection of community health interventions in Spain. The researchers have proposed 3 objectives – (1) first, one to develop and implementation and evaluation tool (2) second, evaluate the impact of implementing the recommendations (3) identify different implementation approaches according to different contexts. They will undertake an Integrative review and expert

	consultation through modified Delphi method to develop the tool. This will be followed by their pilot testing the tool. In the final phase the online version of the tool will be developed.<br><br>Community engagement is an important aspect of health research and knowledge mobilization. There are various models and theoretical frameworks for community engagement available. But less focus is provided in the "make it happen" or implementation part. So, this proposal has merit in filling in the gap of these aspects of community engagement impact assessment. But the proposed protocol is quite unclear to narrate what the researchers want to do. The objectives (1.1, 1.2, and 1.3) are not clearly reflected in the proposed methods and analysis. Seems like the methods will only deliver on the Objective 1.1. The review, Delphi, pilot, and online tool – these all will contribute to the objective of "To develop an implementation and evaluation tool based on the recommendations of NICE guideline NG44".<br><br>The overall writing style need to be revisited. This paper will benefit from tightening up the writing of the paper by making clearly proposing the activities regarding the achieving the objectives.<br><br>For me, another big issue is the lack of critical and in-depth discussion in the "Discussion". Though one may argue that a proposal does not need to have an in-depth discussion section – but there should be discussions on how and why this study will be important to be conducted.<br><br>There seems to be missed opportunity regarding the patient/public involvement. It seems like the community members will be involved as recruiters and knowledge disseminators. But that limits the real aspects of community engagement. The research team need to consider how they can involve community members during the initial phase of this research. I would say that this is surprising that the researchers did not push the envelope a little more to involve community member researchers in this proposal development phase (or, they may have done this – but did not come through their description that clearly).

VERSION 1 – AUTHOR RESPONSE

Reviewer: 1
Dr. Justine Leavy, Curtin University
Comments to the Author:
Evaluating the implementation of community engagement guidelines (EVALUA GPS project): a study protocol.
General comments. Thank-you for asking me to review the manuscript titled as per above and describes the proposed protocol that aims to evaluate the impact of the implementation of the Spanish adapted NICE guidelines. Overall, it is an interesting and under published area. However, I have concerns that a protocol should describe prospective work that has not been commenced and in this paper the integrative review has already been completed and is under review for publication. It is

my understanding that a protocol describes work to be undertaken not work that is partly complete. Perhaps the Phase 1, stage 1 needs to be removed and the manuscript re-written to reflect the work to be undertaken. I assume the Editor-In-Chief will make a decision on this. However, the comments below are provided to strengthen your paper. All the best.
Following comments from the editor, we will not remove phase I but we have shortened that section.

Abstract
Methods page 3 of 13, line 20 suggest you include the proposed dates for the literature review e.g. 2012-2022 if this is to stay in the paper.
Data collection and methods: line 36 suggest you add exploring changes in community engagement intervention to set the context early in the paper.
Thank you for the suggestions, they have both been added to the paper
Introduction:
Could you briefly explain the NICE guidelines and what they cover for readers who may not be familiar with these?
We rephrase the sentence to include an explanation of NICE
Page 5 of 13, line 13-14 Objective 1.2 again I would add community engagement health interventions here so the focus clear, otherwise it could mean any health intervention.
Following your comment later in the text, we changed the terms used in the whole paper to ensure consistency and avoid confusions, so we termed the 'interventions' as 'community-based initiatives', we hope it is clearer now.
Methods and analysis: page 5 of 13 line 26 – is this an analysis of the literature or a review of the literature? I am unsure what an integrative review is exactly. Can the authors provide a definition of how an integrative review is different from a narrative or systematic literature review or a scoping review?
We changed it to review and added a definition and reference about 'integrative' review in the paragraph below

Phase 1: part one: integrative review
This is where I have an issue with the manuscript it describes a '….review will be conducted …..' page 5, line 50. Phase 1: part 2 Page 6 of 13, line 25-27 suggests the review has been completed and under consideration for publication.
Was a systematic search strategy used, e.g. are you following any protocol (eg PRISMA) to ensure rigour?
Page 5 line 56 you comment on peers but did you also engage a reference librarian to refine your search terms? Can the authors comment on this?
We added reference to the published review, so that details about the search strategy, data extraction and analysis can be found directly there. We hope this can help shed light on the issues raised by the reviewer
Page 6 first paragraph the – inclusion criteria is cumbersome, I suggest you simply state criteria 3 and use and/or to include criteria 1 and 2, in one sentence.
Thank you, we deleted the criteria and left the first sentence which included criteria 3
How will you check inter-rater reliability? Will you have a checklist or randomly check selections or exclusions? PRISMA protocol will help with this.
We used the prisma protocol to run the searches, as it is specified in the published article, for which we have now added the full reference
Page 6, line 15 check if ENTREQ needs to be fully described here.
Ok, we added the spelling of the acronym
Phase 1 part two: expert panel through adapted Delphi method
Page 6, line 33, paragraph2 – consider including n = X values to identify how many experts you will recruit. Could the authors comment on using a using snowball method versus selecting experts known to the researchers? See: Naderifar, M., Goli, H., & Ghaljaie, F. (2017). Snowball sampling: A

purposeful method of sampling in qualitative research. Strides in Development of Medical Education, 14(3).
The selection was made by the research team, so we cannot state that a snowball technique was used, as snowball will imply that experts will suggest other people themselves. The selection was made prior to the first round of invitation, combining geographical variability (experts from different regions of Spain) and roles (academic, practitioners, community workers, local organisations' members). We added this information in the text, together with the number of invited people. We also added that the research team has 35 members.
Page 6 , line 45 - how will experts review the tool (Evalguia 1.0) sent a link, then surveyed or interviewed? Can you add some detail here?
We added that it was a word document

Can you authors provide more detail on the 'adapted methodology' you are using and how it differs from traditional Delphi processes? Will round one findings be collated with comments included in round two data collection tools, as is typical for Delphi model. How will consensus be reached? Who will decide, if the experts disagree?

We added an explanation of why 'adapted'.
Phase II: implementation
Page 7 , line 9 - How will the community based interventions be recruited? Are they individual interventions that happen to be starting at the same time or will some be underway already? How will you ensure you capture various types of community based interventions and in various settings – this is especially important if you hope to reach the analysis goals listed on page 9 (Analysis section)
As stated in the inclusion criteria, these will be interventions which are already ongoing (criteria d.). As with the experts, each team member will propose at least one intervention and these will then be discussed and selection made to ensure variability.
Page 7, line 48 - How will you randomise which of the 20 interventions are control interventions (n=4) and which get the full implementation of the tool (n=16)? Can the authors provide some more details
We apologise if this part was unclear, we changed the text to explain how selection was made, we hope this makes it clearer now. We also explicitly mention that we did not randomised the selection of the initiatives.
Intervention
Page 7 line 57, 58 over to page 8 the final sentence in paragraph 1 is incomplete/does not make sense.
Page 8, line 9 you say 'in' two different days it should read on two different days. You may like to have a native English language speaker proof read your paper.
Page 8, line 19, paragraph 2, when explaining the workshop, you state "the Evalguia tool will be implemented" do you mean introduced? Can you please clarify this.
We reviewed the paragraph, and we hope it reads better now. We do mean implemented, as it is tool with questions and spaces for answering and planning ways to improve community engagement.
Data collection
Is the implementation process (Phase II)? If so, please indicate this in paragraph 1
Yes, added
Page 8, line 33 this sentence is awkward consider re-writing 'Written consent will be obtained from each stakeholder prior to data collection commencing.'
Thank you, changed
Page 8, line 41 – consider n=16 intervention groups and n = 4 control groups for clarity and consistency throughout the paper.
We thank you for the suggestion, and we realised that the terms used could have been confusing, so we changed them throughout the paper.
We now used the term 'community-based initiative', as to avoid potential confusion with the term 'intervention' and we then used the term 'intervention' and 'control' for the projects/initiatives in the

phase II explanation. We added ‘workshop’ intervention where needed, to make it clearer.
How will data from community engagement surveys support the development of the tools (As indicated on page 9 – Phase III section) – what questions will you ask? Consider outlining your Domains of inquiry here
We added the information as requested
Page 8, line 56 Would the authors consider semi-structured interviews as better fit for the interviews discussed in stage 1. These interviews are exploring perspectives on potential changes so some structure e.g. around barriers and enablers for the Evalguia tool might be better than unstructured interviews. Can you provide a rationale for unstructured interviews?
Thank you for pointing this out, it was a mistake, we did mean semi-structured interview as we do have a guideline of questions, as stated in the sentence. We changed it to semistructured.
Will qualitative data be recorded, transcribed and will there be any member checking?
Yes, data will be recorded and transcribed, we added the information at the beginning of the paragraph on data collection and more information on analysis at the end.
You state In Stage 3 - 7-9 months after the Evalguia implementation - Do you mean after the workshops and/or intervention has commenced?
Yes, we changed it to evalguia application, hopefully it is clearer with the change in words. We wrote evalguia implementation and not evalguia implementation workshop because there are 4 control groups which will not receive the workshop but will still implement the evalguia. With application we hope it works better.
Data collection includes: Community engagement surveys at 1 month, 4 months and 12 months (n=16 interventions) for all who participated in the workshops (n=15) as well as interviews with intervention manager (n=16) at 1 month, 4 months and 12 months. In addition, an evaluation session/workshop (n=20) and final survey (n=20). Have I understood this correctly? Perhaps include this as a Figure
We had two infographics to invite potential projects, so we translated these into english and we added them in the paper. We hope it helps the readers to understand the different stages
Page 9 line 5 – who exactly are these stakeholders?
We changed it to ‘participants’ and we added more information. We hope it is clearer now.

Phase III: Online tool development
Page 9, line 16 -17 - how will data from community engagement surveys support the development of the online tool
– what questions will you ask? Perhaps outline the variable sets in the Community Engagement Survey tool in Phase II of the study
We added details, following your previous comment on this. Questionnaires and semi-structured interview scripts can be added as annexes if necessary.
Analysis The analysis focus highlights the need for semi-structured interviews rather than unstructured, as described.
What package will you use to analyse the quantitative data and similarly what will be used to analyse the interview qualitative data.
We added information about using Nvivo
Page 9, line 47 onwards – is there any opportunity for triangulation of the data here? Can the authors please comment
We added details about how the analysis will be carried out, by whom and how we will check with the rest of the team. We also modifed the final paragraph of the analysis to include how triangulation will be carried out using both quanti and quali data
Page 9, line 57 You state you aim to enhance translational research – can you give an example or two?
We added the information as requested

General comments for review
Check if headings need be italicised to differentiate from main titles throughout? Eg Should this be

italicised to differentiate from main titles? The headings confuse the flow of this article. (i.e. Methods, Phase II, Intervention).
Page 7 a full stop is required at point d.
Page 8, line 55 - repetition of words in paragraph 4 “asked to answer again to answer” needs deleting National Institute for Health and Care Excellence (NICE) might need to also be written out in full for readers.
We made the suggested changes: italics, full-stop, deletion and nice written in full at the beginning with the introduction.
Figure 1 was hard to read in the version I received. Please update for readability as it looks incomplete with the arrows on the right and all the black space. Is there information missing here?

I am unsure what file was received, this below is what was submitted, we do not see black spaces so I am unsure how to provide an answer to this?

Reviewer: 2
Dr. Tanvir Turin, University of Calgary
Comments to the Author:

This a protocol of study which aims to evaluate the impact of the implementation of the NICE guideline informed Community Engagement in a selection of community health interventions in Spain. The researchers have proposed 3 objectives – (1) first, one to develop and implementation and evaluation tool (2) second, evaluate the impact of implementing the recommendations (3) identify different implementation approaches according to different contexts. They will undertake an Integrative review and expert consultation through modified Delphi method to develop the tool. This will be followed by their pilot testing the tool. In the final phase the online version of the tool will be developed.

Community engagement is an important aspect of health research and knowledge mobilization. There are various models and theoretical frameworks for community engagement available. But less focus is provided in the “make it happen” or implementation part. So, this proposal has merit in filling in the gap of these aspects of community engagement impact assessment. But the proposed protocol is quite unclear to narrate what the researchers want to do. The objectives (1.1, 1.2, and 1.3) are not clearly reflected in the proposed methods and analysis. Seems like the methods will only deliver on the Objective 1.1. The review, Delphi, pilot, and online tool – these all will contribute to the objective of “To develop an implementation and evaluation tool based on the recommendations of NICE guideline NG44”.

We will try to explain with different words the stages of the protocol, and we hope it makes it clearer for the reviewer. The main aim of the project is to evaluate the impact of implementing community engagement guidelines. However, no tools were provided by NICE as to how to translate the theoretical recommendations into practice. Hence, the need to develop an implementation tool which could support the implementation of the adapted guideline’s recommendations in Spain. However, in order to ensure the tool is going to be helpful, we needed to develop a first version (obj. 1.1), and pilot-test it in real-life interventions (obj. 1.2), to make it helpful for practitioners and communities and thus turn it into an online tool available to anyone interested in improving community engagement in their projects (obj. 1.3).

The overall writing style need to be revisited. This paper will benefit from tightening up the writing of the paper by making clearly proposing the activities regarding the achieving the objectives.

For me, another big issue is the lack of critical and in-depth discussion in the “Discussion”. Though one may argue that a proposal does not need to have an in-depth discussion section – but there should be discussions on how and why this study will be important to be conducted.

When writing the paper, we followed the submission guidelines of BMJ for protocol studies: https://bmjopen.bmj.com/pages/authors/#protocol and there is no section about discussion, this is why it has not been included. However, should the editors request it, we would be happy to add a paragraph about ‘discussion’.

There seems to be missed opportunity regarding the patient/public involvement. It seems like the community members will be involved as recruiters and knowledge disseminators. But that limits the real aspects of community engagement. The research team need to consider how they can involve community members during the initial phase of this research. I would say that this is surprising that the researchers did not push the envelope a little more to involve community member researchers in this proposal development phase (or, they may have done this – but did not come through their description that clearly).

We appreciate this comment from the reviewer, and we do agree on the importance of PPI in research. However we would like to point out the current research scenario in Spain, where this study is implemented. Currently, in Spain, PPI is not a specific requirement in most research. The research proposal was submitted to a national competitive call for funding which is traditionally biomedical, and where the language used to present our research proposal needed to follow specific guidelines to ‘pitch’ it to the reviewers, this is why it may seem quite academic in the ways it has been designed. However, as we wrote in the PPI section, the proposal was based on the feedback received by community members from 11 health interventions in the previous project, the AdaptA GPS. The current proposal was accepted and we received funding, so we could not change it afterwards. We did however want to include community members as much as possible, and we added more information in the relevant PPI section, about their involvement also in the delphi method and in the final evaluation session, so we hope it reads better now. We also added the lack of involvement in the design phase as one of the limitations of the study

VERSION 2 – REVIEW

**REVIEWER**	Leavy, Justine<br>Curtin University, School of Public Health
**REVIEW RETURNED**	17-Aug-2022

**GENERAL COMMENTS**	BMJ Open-2022-062383_R1<br><br>Evaluating the implementation of community engagement guidelines (EVALUA GPS project): a study protocol.<br><br>Thank-you for asking me to re-review the manuscript titled as per above and describes the proposed protocol that aims to evaluate the impact of the implementation of the Spanish adapted NICE guidelines. My PhD student Malena Della Bona also assisted with the review.

	Overall the paper is poorly written. The paper lacks clarity and the flow is impeded in places and requires further refinement. AS a protocol it is hard to follow in places which is not ideal. As English is I assume the author's second language it would definitely benefit from someone with English as their first language editing and checking the sentence syntax and grammar throughout. It needs to be much more pithy and a figure or flow diagram might help.<br><br>I hope this is helpful.<br><br>Kind regards<br><br>Assoc Professor Justine Leavy

**REVIEWER**	Turin, Tanvir<br>University of Calgary, Department of Family Medicine, Cumming School of Medicine,
**REVIEW RETURNED**	31-Aug-2022

**GENERAL COMMENTS**	None

VERSION 2 – AUTHOR RESPONSE

BMJ Open-2022-062383_R1

Evaluating the implementation of community engagement guidelines (EVALUA GPS project): a study protocol.

General comments. Thank-you for asking me to re-review the manuscript titled as per above and describes the proposed protocol that aims to evaluate the impact of the implementation of the Spanis h adapted NICE guidelines. Overall, it is an interesting manuscript and an under published area. How ever, the paper lacks clarity and the flow is impeded in places
and requires further refinement. It also appears that not all reviewer comments have been actioned in some places. Our further suggestions are outlined below. All the best with the next stage.

Thank you for your comment and for your suggestions. We apologise if we missed some of your com ments in the first revision. We have now simplified the manuscript based on your comments,
and we hope that this modified version will read better and can
be suitable for publication in the protocol section of the BMJ Open journal.

Abstract

Seems quite long, it could be further refined.

The abstract has been revised and shortened

Introduction

The project objectives are outlined however the aim of the actual paper is now missing or has been d eleted? Please see the last section of the introduction and include a clear aim or esearch question;
a protocol paper needs a clear research aim or statement of what it plans to present.

Being a protocol paper, there was no stated aim of the paper per se
(not even in the first manuscript). However, thank to your suggestion, we added a final sentence at the en dof the introduction so that it states it clearly:

“This paper presents the research protocol for the project EvaluA GPS.”

Methods and analysis:

Please add a reference for the use of a Delphi (adapted) in the methods section.

We realised that the reference was missing when it was mentioned for the first time. We apologise ab out that, thank you for noticing it. We also noticed thatthe reference (it was Reference nº 13, now ref n º10) was not well visible as the indent in the reference list was wrong, but it has now been corrected:

Varela-Ruiz M, Díaz-Bravo L, García-Durán R. Descripción y usos del método Delphi en investigaciones del área de la salud. Investig en Educ medica 2012;1:90–5.

I still have an issue that a protocol should be prospective
and part of this work has already been completed. It seems like a retro-fit.

In the first round of review, we followed the editors’ comment regarding this section on the literature re view, as
Dr. Aldcroft suggested to continue to include it as part of the protocol. We also highlighted to the edito r
(and we apologise that we did not include this information in the response to reviewer file) that two of t he main authors of the manuscript have been on maternity leave in different periods, without anyone c overing for us. This should help explain why the literature review has now been published,
as the two manuscripts were completed in between the maternity leaves and followed different pathw ays and timeframe during the review process, resulting in the literature review now being recently publ ished

Intervention

Consider a diagram or figure here to show the components of the Intervention or add to the Figure in the Reviewer response the specific steps in Phase 2 – Intervention in more detail not just tool and workshop.

We modified Figure 2 to add details about the intervention,
and also changed the text in the manuscript accordingly.

What will the group reflection page 9, line
2 consist of? Is this guided by any reflective practice framework?
As this is a protocol the more specific detail you provide the more robust the process and will improve your implementation.

We changed the word ‘reflection’ to discussion if this seemed confusing,
and we added a sentence to specify more its purpose:

“followed by a group discussion reflection on the results. This will allow participating stakeholders to reflect how community engagement is being currently carried out in their programmes, and to identify areas for improvement.”

Data collection

You are conducting interviews – the fact they are being recorded is secondary, the sentence Page 7, line 48 - needs to be re-written if it is methods it is interviews, ideally the recording, transcribing etc comes after the methods in the analysis section. The informed consent statement should also come before the description of the analysis or remove from here as you have a dedicated section on Ethics at the end of the paper.

We re-structured this section and sentences following your suggestions

Page 11, para 1 - NVivo does not do the qualitative analysis –it is a tool. Can this sentence be reviewed and re- written.

We changed the wording to make it clearer:

“Qualitative analysis will be conducted by two researchers separately, using NVivo Software v12 to aid the analytical process”

Page 11, para 3 consider replacing the word ‘enforce’ the application of evidence based recommendations to *increase* the application or facilitate vs enforce. The paragraph could be reorganised as the additional text about the goals of translational research could come before the first sentences of what your project will deliver or enhance.

However, this paragraph also seems to be more about dissemination of the project findings versus analysis – this content may need to be shifted to later in the manuscript.

This paragraph has been restructured following your suggestions and moved to section **Ethics and dissemination**

Patient and Public Involvement statement

I am not sure what the extra information you have provided adds to this paragraph. It is quite confusing. The figure might be the better addition to describe the phases and stakeholders, and keep the text to a minumum

We modified both figure 1 and 2. In figure 1 we cut down text and added ‘headings’ on top of the diagram, and in Figure 2 we specified the actos involved in each stage, as suggested.

Page 12, para 3 - Dissemination – the design of the interactive tool for readability and inclusive language is possibly best placed as part of the tool design vs

a dissemination strategy. Perhaps a
sub heading that shows this is part of your knowledge translation plan?

We moved the section about the inclusive language at the end of the tool design, as suggested.

General comments for review

I find the writing style quite hard to follow at times. Perhaps a glossary wll help?
I find community based initiatives and 'interventions initiatives' very hard to follow.
I believe that the manuscript needs to be re-worked and be much clearer for the reader.

We re-structured and simplified /changed the text in various sections,
so we hope it now reads clearer.

Consider updating and using the Figure now included in the review comments or a
new flow diagram to show the phases, the stakeholders involved, the number of sites with clear langu age and steps. A glossary might also help.

We changed the figure to add who the stakeholders will be at each stage of the implementation,
and we reworded the manuscript following your suggestions. We hope these changes make it clearer for the reader. We hope with this updated version a glossary is no longer necessary.

Check words that seem to be missing a space, or a missing full
stop or two commas throughout. Eg final dot point of strengths and limitations is missing the full stop. Review formatting of references 17 and 18.

We apologise for this, the extra spaces etc. are due to working online in shared google file
and then downloading in word format for submission. We have now reviewed the format to ensure it di d not happen again.

Good luck.

VERSION 3 – REVIEW

**REVIEWER**	Leavy, Justine<br>Curtin University, School of Public Health
**REVIEW RETURNED**	09-Dec-2022

**GENERAL COMMENTS**	Evaluating the implementation of community engagement guidelines (EVALUA GPS project): a study protocol<br><br>Overall: thank you for the revisions. The authors have diligently set about answering each comment. I still believe a protocol should be prospective, and to this end this paper is not really a protocol for me and/or as per guidelines for protocol preparation and publishing. However, that is now an Editorial decision and out of my hands. All the best with the publication and the project.<br><br>Abstract: has been re-written and made shorter.

	Limitations: include commentary around lack of engagement in the design of the project – however as this is a protocol for what you will do, I believe this should not be included here. Please review.<br><br>Introduction: Now includes the project objectives and outlines what the paper presents. This seems like a basic addition, however describes what the reader can expect to follow in the paper.<br><br>Methods and analysis:<br><br>Page 5 under Phase 1 consider "an expert panel using an adapted Delphi method"… remove the s<br><br>Consider writing 'There will be no exclusion criteria see page 6 last para, versus what is currently there<br><br>Analysis<br><br>The sections for the analysis bring in triangulation as part of the methods. A reference might be worthwhile including where you state triangulation will strengthen the results of the study see page 10 last paragraph. I believe you can remove the words 'of both types of data' from the sentence 'Triangulation of both types of data will strengthen the results of the study, as quantitative data will provide an assessment of changes…" as triangulation infers you are using two types of data to compare and contrast results. Please review.<br><br>Patient and Public Involvement statement<br><br>You have actioned my previous comments, thank-you. I am still not sure what this section adds to the paper, the first part describes or provides context for the new part of the project so I am still not sure if this is where it belongs.<br><br>Ethics – should go under Methods ideally.<br><br>Consider removing the words "As for page 13 start of paragraph. You can write 'The dissemination strategies include xxxxxx. See also below, some final prof reading and a hard edit will be required.<br><br>All the best with the paper and getting it published.<br><br>General

	There are still some clunky sentences that could be further edited throughout the paper.
